# Animal Models of Hepatocellular Carcinoma Prevention

**DOI:** 10.3390/cancers11111792

**Published:** 2019-11-14

**Authors:** Ram C. Shankaraiah, Laura Gramantieri, Francesca Fornari, Silvia Sabbioni, Elisa Callegari, Massimo Negrini

**Affiliations:** 1 Department of Morphology, Surgery and Experimental Medicine, University of Ferrara, 44121 Ferrara, Italy; shnrch@unife.it; 2 Center for Applied Biomedical Research, St. Orsola-Malpighi University Hospital, 40138 Bologna, Italy; laura.gramantieri@aosp.bo.it (L.G.); francesca.fornari2@unibo.it (F.F.); 3 Department of Life Sciences and Biotechnologies, University of Ferrara, 44121 Ferrara, Italy; silvia.sabbioni@unife.it

**Keywords:** hepatocellular carcinoma, prevention, animal models

## Abstract

Hepatocellular carcinoma (HCC) is a deadly disease and therapeutic efficacy in advanced HCC is limited. Since progression of chronic liver disease to HCC involves a long latency period of a few decades, a significant window of therapeutic opportunities exists for prevention of HCC and improve patient prognosis. Nonetheless, there has been no clinical advancement in instituting HCC chemopreventive strategies. Some of the major challenges are heterogenous genetic aberrations of HCC, significant modulation of tumor microenvironment and incomplete understanding of HCC tumorigenesis. To this end, animal models of HCC are valuable tools to evaluate biology of tumor initiation and progression with specific insight into molecular and genetic mechanisms involved. In this review, we describe various animal models of HCC that facilitate effective ways to study therapeutic prevention strategies that have translational potential to be evaluated in a clinical context.

## 1. Introduction

Liver cancer is the sixth most common cause of cancer worldwide and accounted for nearly 780,000 deaths or 8% of all cancer related deaths as of 2018 [1]. Globally, liver cancers are more common in men than women, with a ratio of 3:1, and burden of disease is predominant in countries like China, Mongolia, Southeast-Asia and Africa (Sub-Saharan, Western and Eastern), accounting for nearly 83% of global liver cancer diagnosis [2]. Global liver cancer associated mortality rates follow the same trends as incidence and prevalence of the disease [1,3]. Disability-Adjusted Life Year (DALY) is an adequate measure to quantify burden of disease from morbidity and mortality. Liver cancer caused ~21 million DALYs in 2016, with Years of life lost (YLLs) accounting for 99% of DALYs [2]. Along with lung cancers, liver cancers amount to the highest YLLs in the general population. The aggressive nature and poor survival of liver cancer makes it an important public health issue worldwide [4].

Primary liver cancers include hepatocellular carcinomas (HCC), carcinomas of gallbladder, intrahepatic cholangiocarcinomas (ICC) and extrahepatic cholangiocarcinomas. The vast majority (85 to 90%) of primary liver cancers are HCC with ICC accounting for most of the other subtypes. The burden of HCC is not evenly distributed worldwide and this disparity in HCC occurrence not only depends on low sociodemographic indices of these regions but also on race and ethnicity. For example, in the United States of America, a country with high sociodemographic index, incidence of HCC in Asians was 2 fold higher than white Hispanics and 4 fold higher than Caucasians [5]. The reasons for this ethnic disparity might include differences in acquisition time of risk factors for chronic liver disease leading to HCC. 

## 2. Hepatocellular Carcinoma Risk Factors

About 80 to 90% of all HCC occurs within a background of chronic liver disease and cirrhosis. Broadly, risk factors for cirrhosis in HCC patients can be dichotomised into viral and non-viral factors. Major viral risk factors include hepatitis B virus (HBV) and hepatitis C virus (HCV) infections and are a global health problem resulting in chronic hepatitis that can progress to liver cirrhosis and HCC. Large population-based studies in chronic hepatitis B (CHB) and chronic hepatitis C (CHC) patients have identified high serum HBV DNA, HCV RNA viral load respectively, as independent risk factors for developing HCC [6,7].

HBV, a member of the Hepadnaviridae family, is a double-stranded DNA virus. Humans are the only natural hosts of HBV and its tissue tropism is limited to liver and particularly to hepatocytes. The virus is transmitted through contact with blood or other body fluids and vertically from infected mother to the child. Once infected, manifestation of symptoms is age-dependent in acute stage. In children, most infections are clinically silent. In adults, up to 30% of cases show transient jaundice and flu-like symptoms and in up to 70% of cases an increase in serum transaminases are documented as subclinical hepatitis [8]. Progression of CHB is often not apparent until cirrhosis or end-stage liver disease is diagnosed. Current clinical practice guidelines suggest utilizing a combination of hepatitis B e-antigen (HBeAg), hepatitis B surface antigen (HBsAg) and serum HBV DNA levels to assess risk of HCC in CHB patients [9]. At present, there are no therapies to eliminate HBV infection. Management of chronic disease is aimed at slowing the process of liver decompensation and to decrease systemic viral load. Long-term administration of nucleos(t)ide analogues like entecavir, tenofovir disoproxil and tenofovir alafenamide are treatment of choice, since they inhibit reverse transcription and restrict HBV replication in majority of cases. Pegylated interferon-alfa might be considered in early stages of CHB and is associated with severe side effects. Typically, treatment is initiated at HBV DNA > 2,000 IU/ml and elevated levels of liver transaminases [10]. 

HCV, a member of the Flaviviridae family, is an encapsulated positive-sense single-stranded RNA virus. HCV spread is exclusively blood borne. In majority of cases, acute HCV infection is asymptomatic and only 15% of cases present with jaundice, elevated transaminases. In adults, 55–85% cases develop CHC, with clinical course ranging from modest histopathological changes to fulminant hepatitis that progresses to liver fibrosis, cirrhosis and HCC. However, the progression of CHC to HCC occurs over several decades. Treatment regimens for HCV infection are aimed at a curative outcome. Virological response means the virus is not detected upon treatment and efficacy of treatment is measured in terms of sustained virological response (SVR). With direct-acting antiviral (DAA) therapies becoming mainstay treatment options for HCV, outcome of HCV treatment has been optimistic. Several DAA agents (sofosbuvir, simeprevir, velpatasvir, glecaprevir, asabuvir, ombitasvir) have been approved and used alone or in combination with significant SVR rates of greater than 90% after 8 to 12 weeks of treatment for almost all HCV genotypes [11]. It would be interesting to see long term effects of DAA therapies on HCC risk as high rates of SVR and eradication of HCV will have tremendous impact on chronic liver disease progression and HCC incidence. 

Non-viral risk factors for HCC include cirrhosis from various causes (e.g., alcoholic cirrhosis); obesity, diabetes mellitus type-2 (DM2); inherited errors of metabolism such as hereditary hemochromatosis, porphyria cutanea tarda, and alpha-1 antitrypsin deficiency; Wilson’s disease [12]. Environmental exposure to aflatoxin, a natural product of *Aspergillus* fungus and excessive alcohol intake are other risk factors for HCC [13]. Studies evaluating incidence risk of HCC in alcoholic cirrhosis are confounded by the presence of other concomitant risk factors like CHB, CHC, obesity and DM2 [14]. Furthermore, metabolic disorders (e.g., obesity, DM2, impaired glucose metabolism, metabolic syndrome, non-alcoholic fatty liver disease (NAFLD)) are associated with increased risk of HCC [15]. In the past decade numerous epidemiological studies have shown obesity and DM2 to be a risk factor for cancer [16,17]. In the USA, about 20% of all cancer deaths in men and 14% in women were documented in individuals with body mass index (BMI) ≥ 30. The relative risk (RR) of dying due to liver cancer in individuals with BMI ≥ 35 was highest among all cancers in men (RR = 4.52) [18]. Similar to obesity, epidemiological association studies have documented an increased risk for HCC in DM2 patients [19,20].

## 3. Hepatocellular Carcinoma Prevention

HCC and cirrhosis are the major life-limiting consequences of progressive chronic fibrotic liver disease, caused by any etiology. Although complete HCC tumor resection or ablation at early stages of disease is effective, underlying tumor conducive tissue microenvironment in the remnant liver could give rise to recurrence of tumors that progress to unresectable advanced-stage disease in majority of patients. Once tumors progress to advanced-stage, current approved medical therapeutics provide meagre survival benefit [21,22]. 

Thus, early detection and prevention of liver fibrosis progression to HCC would be an effective strategy to ameliorate prognosis of patients. A bi-annual HCC screening for early detection of tumors, as recommended by clinical practice guidelines, is an option [23]. Cancer prevention strategies can also represent a valuable mean to decrease HCC burden in at-risk population. 

Preventive interventions are aimed at eradicating risk factor affliction and interrupting cell signaling pathways that promote carcinogenesis. Primary prevention focusses on prophylactic elimination of HCC risk at an early stage before onset of any liver disease. These interventions could include lifestyle modifications to combat obesity, intake of whole grains in diet is associated with reduced risk of HCC [24], universal newborn immunization programs against HBV in the early nineties and screening for HCV before blood transfusions during the same time-period have been effective in preventing viral hepatitis infections. As a consequence of these public health measures, hepatitis virus associated HCC risk has greatly decreased in general population [25,26]. Several decades of research in large cohort studies have associated regular aspirin use with reduced HCC risk [27,28]. 

Secondary prevention encompasses chemointervention to prevent occurrence of HCC or progression of pre-neoplastic hepatic foci to neoplasia in patients already exposed to aetiological risks [29]. Owing to the long latency period between liver fibrosis and formation of tumors, HCC secondary prevention makes for an attractive health measure. However, tumor heterogeneity and incomplete understanding of mechanisms involved in neoplastic transformation in HCC predominantly form the roadblocks to development of chemoprevention strategies [30]. Added to that, potential chemoprevention agents would ideally have to be inexpensive and relatively tolerable in terms of toxicity to be justified for long term use in clinical practice. Several epidemiological association studies have indicated towards potential chemoprevention agents. Metformin use in DM2 population [31,32,33] and statin use in cirrhotic patients [34] has been associated with lower incidence of HCC. An ongoing phase 2 clinical trial is evaluating simvastatin as a chemoprevention agent in cirrhotic patients (NCT02968810) but similar clinical trials with metformin (NCT02319200-Terminated, decision of investigator, NCT02306070-withdrawn, insufficient funding) were not completed. Potential reduction of HCC risk post management of chronic hepatitis B and C infection with nucleos(t)ide analogues and DAA agents, respectively, had long been unclear. However, a European multi-center cohort study reported that post entecavir/tenofovir therapy, risk of HCC occurrence beyond 5 years decreased in younger patients without cirrhosis [35]. Interestingly, preservation of liver function in the long-term improved over all survival in HCC patients after HCV eradication regimens with DAA agents [36]. 

Tertiary prevention includes chemointervention to prevent recurrence of HCC after initial resection or liver transplantation. Clinical trials evaluating chemopreventive agents in a tertiary prevention setting might be more appealing due to shorter timeframe of research studies in both academic and pharmaceutical industry setting. 

**Figure 1:**
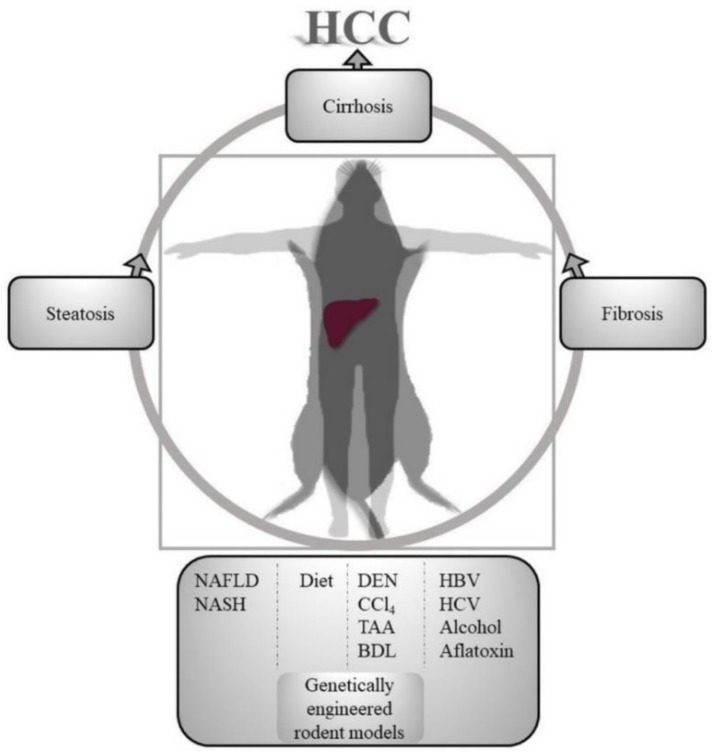
Translational aspects of hepatocellular carcinoma (HCC) prevention animal models. Various fatty liver induction, immunogenic, hepato-carcinogenic, hepato-toxic methods are used in rodents to model human hierarchical progression of liver injury to HCC. NAFLD, Non-alcoholic fatty liver disease; NASH, non-alcoholic steatohepatitis; DEN, dimethylnitrosamine; CCl_4_, carbon tetrachloride; TAA, thioacetamide; BDL, bile duct ligation; HBV, hepatitis B virus; HCV, hepatitis C virus.

To overcome these challenges, pre-clinical animal models of HCC prevention empower researchers to study complex and dynamic tumor pathophysiology systems. Particularly, in the area of precision cancer prevention, these animal models are invaluable since in-vitro systems cannot be configured to reflect tissue or organ systems. Chronicity of tumorigenesis process in HCC further limits in-vitro models. However, the animal models might not completely resemble the human disease process, leading to false discovery of chemoprevention targets and biomarkers; these approaches may be improved by more sophisticated modelling strategies (Figure 1) [37].

## 4. Induction Models of Chronic Liver Injury and HCC

Several methods are utilized to induce chronic liver injury (CLI) in rodent models. These can be broadly categorized into chemical and diet mediated induction of CLI. Assorted genotoxic and hepatotoxic chemicals such as dimethylnitrosamine (DMN), diethylnitrosamine (DEN), thioacetamide (TAA), carbon tetrachloride (CCl_4_), ethanol and aflatoxin are administered to induce HCC in drinking water, by intraperitoneal injection, oral gavage or gaseous inhalation. 

DEN is a genotoxic carcinogen that causes liver tumors in rodents in varied manner depending on age, gender, dosage protocol and strains used. DEN mediated carcinogenesis animal models are frequently used to investigate chemopreventive agents. In mice, chronic DEN additionally results in various gastrointestinal tumors [38,39]. DEN is metabolised in the liver to electrophilic ethyldiazonium ion and together with reactive oxygen species induces DNA damage [40]. This DNA damage in actively proliferating hepatocytes initiates genome aberrations that eventually lead to tumorigenesis. Hence, a single bolus intraperitoneal injection of DEN in 10 to 15-day old neonatal mice, causes liver tumors. Several phytochemicals have been previously described as potential HCC chemopreventive agents in DEN models [41]. These plant derivatives include curcumin, a low molecular weight polyphenol derived from the root of *Curcuma longa* has been extensively studied to have hepatoprotective properties. In both mouse and rat models, curcumin was shown to inhibit DEN-induced expression of oncogenic HRAS, focal dysplasia and hepatocarcinogenesis [42,43,44]. Another phytochemical, resveratrol, abundant in peanuts and grapes, could interfere with DEN-induced hepatocarcinogenesis by activating apoptotic pathways in male Wistar rats [45]. The most abundant of green tea polyphenols, epigallocatechin gallate (EGCG) administration could decrease preneoplastic glutathione S-transferase placental form (GST-P)-positive foci in rodent models [46]. 

Furthermore, several metabolic and signaling pathway regulators have been studied as chemoprevention strategies in DEN rodent models. The following are prominent illustrations. Inhibition of de novo lipid biosynthesis by targeting sterol regulatory element-binding protein (SREBP) pathway prevents tumorigenesis in DEN mouse model [47]. Metformin, a dimethylbiguanide that is frequently used as an anti-diabetic agent, could prevent hepatocarcinogenesis by suppressing hepatic progenitor cell activation and advanced glycation end products in chronic DEN-induction rat models [48,49]. Meta-analysis of genome-wide transcriptome profile from more than 500 human cirrhosis patients, identified co-regulated gene modules that could drive HCC risk with lysophosphatidic acid (LPA) pathway as key target for chemoprevention. Autotaxin (ATX) and Lysophosphatidic acid receptor 1 (LPAR1) are key upstream members of LPA pathway. Targeting ATX and LPAR1 with respective inhibitors (AM063 and AM095) in chronic DEN-induced rat HCC model, could attenuate liver fibrosis and prevent HCC via suppression of RhoA and ERK pathways [50]. DEN-induced initiation of carcinogenesis combined with choline-devoid methionine-deficient (CMD) in F344 rats resembled human NAFLD with fatty liver changes and GST-P positive preneoplastic hepatocyte foci driven by activation of transcription factor Nrf2. Genetic ablation of Nrf2 in this nutritional rat model of liver carcinogenesis, prevented hepatocarcinogenesis initiation despite presence of CMD-induced cirrhotic changes, thereby underlying the rationale for developing Nrf2 inhibitors as chemoprevention agents [51]. Exhaustive description regarding DEN pathogenic mechanism and protocols are described in detail elsewhere [52].

Liver fibrosis can be induced in rodents by surgical intervention (bile duct ligation) or more commonly, by administration of hepatotoxins. CCl_4_ is a hepatotoxin that induces liver injury and leads to panlobular parenchymal liver fibrosis [53]. CCl_4_ challenged mice are ideal tools for studying hepatic remodeling [54]. In the liver, cytochrome P450 2E1 (CYP2E1) is a key metabolizing enzyme in mediating the hepatic damage following CCl_4_ exposure [55]. Hence, CCL_4_ model is often utilized to study anti-fibrosis treatment strategies. Epidermal growth factor (EGF) and its receptor expression are upregulated in cirrhotic liver disease [56]. Therefore, EGFR inhibitors are an attractive chemopreventive agents. Erlotinib, an EGFR inhibitor, could inhibit progression of liver fibrosis in DEN, BDL rat and CCl_4_ mouse models. These antifibrotic effects resulted in abrogation of HCC development in DEN rats [57]. However, in advanced HCC setting, addition of erlotinib to first-line sorafenib treatment did not improve overall survival in a phase III clinical trial (NCT0901901) [58]. A pilot phase I/II clinical trial with erlotinib for prevention of HCC in cirrhosis patients is ongoing (NCT02273362). 

Non-alcoholic steatohepatitis (NASH) is emerging as a major risk factor for HCC development owing to diet and lifestyle choices of modern life [59]. NASH is characterized by chronic hepatitis, lobular inflammation and associated metabolic disease. To study chemoprevention strategies in progression of NASH to HCC, STAM mouse model has been proposed [60]. In this mouse model, metabolic disease is induced by low-dose streptozotocin in neonatal C57BL/6J and providing high-fat diet (HFD) at 4 wks of age. As a consequence, STAM model mice develop NASH at 8 wks, HCC at 16–20 wks and effectively recapitulates NASH to HCC progression in human patients. Utilizing STAM model, sodium-glucose co-transporter 2 (SGLPT2) inhibitor, canagliflozin, was shown to attenuate steatohepatitis, liver fibrosis and tumorigenesis [61]. 

## 5. Genetically Engineered Animal Models of HCC Prevention

With the advent of genome-editing technologies, genetically engineered animal models of HCC have been developed. However genetically engineered rats lag behind the mouse models. Most of these genetically engineered mouse models (GEMs) are engineered using concepts derived from reverse genetics, where in, frequent gene mutations or aberrant expression in HCC patients are introduced with the intent of generating spontaneous HCC development phenotypes [62]. Class II germinal center kinases MST1/2 have been shown to act as tumor suppressors and knockout mice of these genes developed spontaneous HCC, underlying the importance of Hippo-Lats-Yorkie signaling in regulating tumorigenesis [63]. The PTEN lipid phosphatase, acts as a tumor suppressor gene by regulating PI3-kinase signaling pathway. PTEN-null mice developed steatohepatitis and histological human NASH features at 10 months, and progression to HCC took about 18 months [64]. In this mouse model, novel STAT3 inhibitor C188-9 improved NASH and liver function along with reduced tumor development. These hepatoprotective effects of C188-9 were attributed to downregulation of TREM-1 and interferon-γ inducible genes [65]. Additionally, maintaining PTEN-null mice on HFD for 40 weeks resulted in exacerbation of inflammation, hepatic ballooning, liver fibrosis and increased NAFLD activity score. These detrimental effects promoted by HFD was attributed to hypercholesterolemia and VEGF mediated augmented angiogenesis. Addition of ezetimibe, a cholesterol lowering agent, to HFD provided a hepatoprotective effect by attenuated steatohepatitis and suppressed tumor formation via inhibition of angiogenesis. However, ezetimibe had no effect on tumorigenesis in PTEN-null mice fed on normal diet [66]. Several studies have highlighted that activation of toll-like receptor 4 (TLR4) in tumors and stromal cells effects tumor progression and evasion from immune surveillance [67,68]. In accordance with these observations, TLR4 inhibitor, resatorvid, was shown to prevent liver tumorigenesis in mice with hepatic deletion of PTEN [69]. 

Deficiencies in autophagy, a physiological process by which cells degrade cytoplasmic components within lysosomes, are associated with increased tumorigenesis [70]. ATG5 and ATG7 are essential for autophagic process and knockout mice of these genes developed liver tumors spontaneously [71]. More than three decades ago similar observations of liver tumorigenesis were observed by overexpressing c-MYC and TGFα in mice [72,73]. 

Hepatic stellate cells (HSCs) activation is a major contributor to liver fibrosis. Homodimer PDGF-CC stimulates DNA replication in HSCs [74]. To assess the potential role of PDGF-C in driving liver fibrosis, Pdgf-c expressing transgenic (Pdgf-c Tg) mice were generated [75]. These mice develop liver fibrosis and steatosis by 9 months of age, followed by HCC at 12 months (~ in 80% of the mice). Liver fibrosis in Pdgf-c Tg mice is perpetuated by activated HSCs resulting in transformation of these cells to myofibroblast-like cells and promotes EMT transition [76]. Peretinoin, a member of the acyclic retinoid family with vitamin-A like structure when administered at 0.06% in diet for 44 weeks, could prevent liver fibrosis and tumors in Pdgf-c Tg mice by inhibiting angiogenesis and canonical Wnt/β-catenin signaling [77]. However, hypervitaminosis A-induced osteopenia was encountered in 5% of the experimental mice. A multicenter randomized study reported that branched chain amino acids (BCAA) improved liver function and decreased Child-Pugh score in advanced cirrhosis patients [78]. To study hepatoprotective mechanism of BCAA, Pdgf-c Tg mice were fed basal diet supplemented with 3% BCAA for 20 weeks. BCAA intervention decreased incidence of fibrosis and tumors in the liver but did not have any significant effect on liver function. These hepatoprotective effect of BCAA was attributed to mTORC1-mediated inhibition of pro-fibrotic TGF-β1 signaling [79].

## 6. MircoRNAs and HCC Prevention Models

MicroRNAs (miRNAs) are a class of small non-coding RNAs of 19–24 nt in length and play significant gene-regulatory role by binding to target mRNAs of multiple protein-coding genes. Hence anti-miRs and miRNA mimics make for potential therapeutic agents. MiRNA dysregulation is a key factor that has long been studied in formation of tumors [80]. Understanding of the important differentially expressed miRNAs has been harnessed to model transgenic mice that spontaneously develop liver tumors. 

MiR-214 has been reported to be upregulated during progression of hepatic fibrosis and in chronic hepatitis B and C patients [81]. Pdgf-c Tg mice treated at 9 weeks of age (liver fibrosis) and 32 weeks (HCC) with locked nucleic acid anti-miR-214 showed a marked reduction in fibrosis and tumor incidence and these effects were attributed to repression of EGFR and TGF-β signaling pathways [82]. miR-122 is an abundant liver-specific miRNA that regulates hepatic homeostasis and functions as a tumor suppressor [83]. In human NASH patients, miR-122 expression is downregulated in the liver [84]. Additionally, decreased expression of miR-122 corresponds with metastasis and poor outcome in HCC patients [85]. Germline deletion of miR-122 locus in mice (miR122-KO) resulted in hepatic inflammation that progressed to fibrosis, chronic steatohepatitis with age and eventually by 10–15 months of age, these mice developed HCC [86]. In these mice, hepatic inflammation was attributed to chemokine Ccl2, that is overexpressed in HCC [87], and subsequent infiltration of CD11b^hi^Gr1^+^ inflammatory cells. Replenishing miR-122 via adeno associated virus delivery, suppresses liver tumorigenesis in non-inflammatory tet-o-MYC;LAP-tTA HCC mouse model [86,88]. 

Mir-21 and mir-148a are frequently dysregulated in HCC and are associated with poor prognosis [80,89]. Translating these observations in clinical specimens, anti-miR-21and mir-148a-minetics were shown to abrogate liver fibrosis and progression to HCC in hepatic PTEN null mice. HCC preventive effects of targeting miR-21 were attributed to depletion of CD24^+^ liver progenitor cells, S100A4^+^ tumor associated stromal cells and NOTCH pathway [90]. While miR-148a-mimetics induced hepatocyte differentiation and suppressed NOTCH activity resulting in prevention of tumor development. Furthermore, targeting NOTCH with an inhibitor OR4929097 resulted in similar hepatoprotective effects as seen with miR-148a-mimetics, hence highlighting NOTCH as a promising target for HCC chemoprevention [91]. 

MiR-221 is one of the most frequently up-regulated miRNAs in HCC patients and is regarded as an oncogenic miRNA [80,92]. Taking these observations into account, we developed liver-specific miR-221 overexpressing transgenic mouse model (TG221) that exhibited strong predisposition to development of spontaneous liver tumors at 9–12 months of age. In this model, liver tumors are accompanied by hepatomegaly, steatohepatitic changes, hepatocyte degeneration marked by enlarged dysplastic nuclei and focal coagulative necrosis. At the molecular level, significant inhibition of Cdkn1b/c, miR-122 and miR-199 was observed [93]. Autochthonous tumorigenesis in these mice could be accelerated by low dose DEN i.p in 10–15-day old mice and by chronic CCl_4_ challenge for 14 weeks in adult mice. The latter providing an added advantage of liver tumors that developed in cirrhotic liver background. Aiming to test the HCC prophylactic potential of miRNA-based molecules, we demonstrated that miR-221 inhibition and miR-199 replacement were also able to prevent malignant transformation of nodular lesions in CCl_4_-treated TG221 mice, without inducing toxicity [94]. In both settings, biological outcomes were accompanied by molecular regulation of miR-221 and miR-199 target proteins. Hence, confirming the pivotal importance of these miRNAs in regulation of cancer-related pathways.

Additionally, progression of fibrosis could be studied in TG221-CCl_4_ mouse model. Early or first signs of portal liver fibrosis was observed after 3 weeks of initiation CCl_4_ challenge and gradually progressed to cirrhosis by 14 weeks. TG221-CCl_4_ made for a utilitarian model, whereby early chemointervention strategies could be studied. To this end we studied chemopreventive effect of early metformin intervention. TG221-CCl_4_ at 3 weeks were treated with daily metformin treatment, that was continued until the experimental endpoint (10 weeks after cessation of CCl_4_ challenge). Metformin could effectively attenuate progression of liver fibrosis and as a consequence prevent formation of liver tumors. This hepatoprotective effect of metformin was largely mediated by suppressing hepatic stellate cell activation, PI3K/AKT and EMT pathways in the liver [95]. 

## 7. Conclusions

Undeniably, most cases of HCC develop in the setting of chronic liver disease, characterized by cirrhosis, liver fibrosis and steatosis. GEMs described above cater to genetic perturbation in oncogenes and tumor suppressor genes. Although these models are invaluable in studying molecular and signaling pathway interactions during tumor initiation and progression, their utility is limited by long incubation times and inability to recapitulate underlying liver disease hierarchy. Several toxins, carcinogens and modified diets are combined with GEMs to accelerate tumorigenesis and closely resemble underlying human liver disease. Evidently, no one rodent model could recapitulate the process of human hepatocarcinogenesis in its entirety. Suppression of PTEN expression and activity appears to be a paramount molecular event in HCC of various aetiologies [96,97]. Hence, reverse engineering to reflect liver specific PTEN downregulation as seen in PTEN-null and TG221 (PTEN being a major miR-221 target) mouse models combined with chronic liver injury induction could be useful models to test precision chemoprevention agents. Notably, repeated low dose DEN induction in rats was shown to concur with HCC-risk gene expression signature [98]. There are adequate pre-clinical evidences regarding chemopreventive agents that have shown promise and could be translated to clinical application (Table 1). However, considering long latency period involved in progression of liver fibrosis or cirrhosis to HCC, prevention directed clinical trials would be spread over a longer duration and cohort sample size might need to be large to determine smaller effect size. To overcome these challenges, potential chemoprevention agents have been suggested to be introduced into tertiary prevention wherein prevention of recurrence could be tested [99]. Finally, non-invasive biomarkers of progression of HCC from early stages of fibrosis to cirrhosis and eventual frank HCC, could define appropriate clinical endpoint to assess HCC prevention drugs [100]. Advancement of clinical availability of chemopreventive agents could significantly improve the prognosis of chronic liver disease patients and decrease HCC incidence and mortality.

## Figures and Tables

**Table 1:** HCC prevention rodent models and intervention strategies.

Animal Model		Induction	Chronic Liver Injury	Pathogenesis	Preventive Intervention	Ref.
Rodent	Strain					
RAT	(Wistar)	DEN	Repeated weekly IP	Fibrosis (8 wks) progress to cirrhosis (12 wks) and HCC (18 wks)	Metformin, AM063, AM095, erlotinib, gefitinib	[48,50,57,101]
	(F344)	DEN	CMD diet	Steatosis progresses to steatohepatitis and fibrosis with GST-P +ve pre-neoplastic after 17 wks	NRF2 KO	[51]
	(Wistar)	BDL		Fibrosis after 3 wks	Erlotinib	[57]
MOUSE	(C57BL/6J)	DEN		Lipid biosynthesis (SREBP pathway) regulates HCC initiation and development	Gp78 KO/SCAP KO/Betulin	[47]
	(A/J)	CCL_4_	Repeated thrice weekly PO	Fibrosis after 18 wks	Erlotinib	[57]
	(C57BL/6J)	STZ+HFD	Neonatal low-dose STZ SQ & HFD at 4 wks	NASH by 8 wks progress to HCC by 16-20 wks	Canagliflozin	[60,61]
	AlbCrePten^flox/flox^			NASH- hepatomegaly, steatosis, inflammation, fibrosis and progress to HCC	C 188-9	[64,65]
	AlbCrePten^flox/flox^		HFD for 40 wks	NASH-related cirrhosis and HCC with hypercholesterolemia	Ezetimibe	[66]
	*Pten^loxP/loxP^*; *Alb-Cre^+^*			HCC by 8–9 months	Resatorvid, anti-miR-21, miR-148a,	[69,90,91,102]
	PDGF-C transgenic			Fibrosis and steatosis by 9 months progress to HCC by 12 months	Peretinoin, BCAA, LNA-antimiR-214	[75,77,79,82]
	miR-221 transgenic		Short CCL_4_ inhalation cycles for 14 weeks. Phenobarbital in drinking water.	Progression from fibrosis to cirrhosis and HCC	Anti-miR-221 oligonucleotides, miR-199a-3p mimics	[94]
	miR-221 transgenic		CCL_4_ PO repeated thrice weekly for 14 weeks	Early fibrosis progress to cirrhosis and HCC	Metformin	[95]
	Tet-o-MYC; LAP-tTA			MYC overexpression leads to liver tumors by 15 wks. No fibrosis/cirrhosis	miR-122	[86,88]
	C57BL/KsJ-+Lepr *^db^* /+Lepr *^db^*		DEN in drinking water for 2 wks	DEN initiates tumorigenesis that is promoted by obesity and diabetes. Chronic inflammation and steatosis progress to HCC	Tofogliflozin	[103]

DEN, diethylnitrosamine; CMD, choline-devoid methionine-deficient; CCL_4_, carbon tetrachloride; BDL, Bile duct ligation; HCC, Hepatocellular carcinoma; NASH, non-alcoholic steatohepatitis; GST-P, glutathione S-transferase placental form; SREBP, sterol regulatory element-binding protein.; SCAP, SREBP cleavage-activating protein; HFD, high fat diet; BCAA, branched chain amino acids; LNA, locked nucleic acid; PDGF-C, Platelet-derived growth factor C; IP, intraperitoneal injection; PO, oral gavage; STZ, streptozotocin; SQ, subcutaneous.
